# A computational chemistry-based approach to optimizing PD-1/PD-L1 inhibitors

**DOI:** 10.3389/fchem.2024.1533541

**Published:** 2025-01-14

**Authors:** Meijuan Zhai, Shiliang Ji, Haoran Hu, Yongjie Wu, Yi Shi, Ruifang Zhu, Yiguo Jiang, Yang Yang

**Affiliations:** ^1^ Department of pharmacy, The Affiliated Suzhou Hospital of Nanjing Medical University (Suzhou Municipal Hospital), Gusu School, Nanjing Medical University, Suzhou, China; ^2^ Suzhou Research Center of Medical School, Suzhou Hospital, Affiliated Hospital of Medical School, Nanjing University, Suzhou, China; ^3^ State Key Laboratory of Pharmaceutical Biotechnology, School of Life Sciences, Nanjing University, Nanjing, China

**Keywords:** non-small cell lung cancer, PD-1 inhibitor, PD-L1 inhibitor, molecular docking, computer-aided drug design

## Abstract

**Introduction:**

To design effective small molecule inhibitors targeting the immune checkpoint PD-1/PD-L1 and to explore their inhibitory activity.

**Methods:**

In this paper, a total of 69 PD-1/PD-L1 inhibitors with the same backbone were searched through opendatabases, and their docking mechanism with PD-L1 protein was investigatedby molecular docking method, and the active conformation of the inhibitors was explored. The biological activity of the four newly designed inhibitors was also evaluated using ELISA.

**Results:**

The most active molecule 58 in the dataset formed six hydrogen bonds with Phe67, Val55, Ile116 and Tyr123, while the second most active molecule 34 formed five hydrogen bonds with Phe67 and Ala121, both of which formed π-π stacking interactions with Tyr56. The analysis of the inhibitor docking results determined that the residues Tyr123, Gln66, Thr20, Met115, Asp122 and Ile116 had the greatest influence on the active conformation of the inhibitor. ELISA assays suggested that the four novel inhibitors designed had high inhibition rates, with the inhibition rate of compound N2 being as high as 68.53%.

**Discussion:**

In this paper, we have designed and synthesized various PD-1/PDL1 inhibitors, which provide a basis for drug discovery targeting the PD-1/PDL1 signaling pathway.

## 1 Introduction

Lung cancer is a malignant tumour originating from the trachea, bronchi and lung, with the second-highest incidence rate worldwide and the highest mortality rate among all malignant tumours. Lung cancer has been one of the most common malignant tumours in clinical practice today (Heinzerling et al., 2021; Seguin et al., 2022). Currently, surgery, radiotherapy and targeted therapy are commonly used in clinical practice, but the inescapable problems of drug resistance, toxic side effects and poor treatment outcomes have prevented these approaches from fundamentally improving the survival time of patients (Hamilton and Rath, 2019; Zhou et al., 2021). This poses the whole new challenge in the search for other effective treatment methods and tools. Immunotherapy is emerging as one of the most promising therapeutic approaches compared to traditional treatment modalities (Bader et al., 2020).

Immunotherapy is a method of treating disease by artificially interfering with immune functions and processes. The immune checkpoint programmed death receptor-1 (PD-1) and programmed death ligand 1 (PD-L1) are a self-protective measure of the body and have become a focal point in the treatment of non-small cell lung cancer as one of the important pathways mediating this disease. PD-L1 immune checkpoint inhibitors can effectively treat cancer by inhibiting or blocking the binding between PD-1 and PD-L1, allowing the patients to regain its immune function to recognize and kill cancer cells, thus achieving the satisfactory therapeutic effect. The safety of the use of these inhibitors and other great advantages have changed the direction of treatment for non-small cell lung cancer (Huang et al., 2021; Lin et al., 2020).

As monoclonal antibodies have poor permeability, poor oral absorption and immune-related adverse effects in clinical applications, small molecule inhibitors have significant advantages over biomolecules in terms of less immunogenic side effects, better penetration and lower cost. It is essential to design and optimise novel PD-1/PD-L1 small molecule inhibitors at the receptor structural level to enhance drug-protein interactions. However, there is limited research on the mechanism of interaction between these inhibitors and PD-1/PD-L1 proteins, which poses a challenge for the development of new drugs. There are currently no investigational drugs available for this target in China. Since experimental process is long and costly, computer-aided drug design has received a lot of attention in recent years and can be a good solution to these problems. The receptor-based structure design is one of the most important approaches to drug design and molecular docking is a powerful tool for investigating the mechanism of action between small molecule drugs and their receptors (Fan et al., 2015; Liu et al., 2021).

In summary, this study is based on computational chemistry and uses molecular docking to determine the docking binding mechanism and the composition as well as the details of the active cavity of this class of small molecule inhibitors to PD-1/PD-L1 proteins. The results obtained were used to design and optimize the local structure of PD-1/PD-L1 inhibitors, which can provide theoretical guidance for the development of novel PD-1/PD-L1 inhibitors.

## 2 Material and methods

### 2.1 Datasets

**FIGURE 1:**
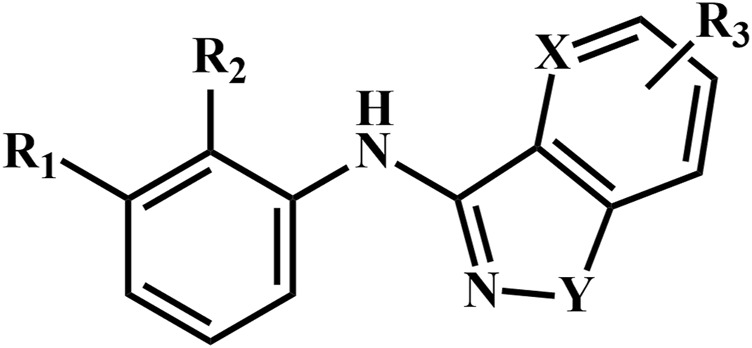
Structural characteristics of 69 inhibitor molecules in the database.

A total of 69 novel small molecule inhibitors of PD-1/PD-L1 with the same backbone, all of which have been shown to have significant inhibitory activity and summarised in this paper. The structural and activity data of these molecules were obtained from the laboratory of Z.Q. Feng (Dai et al., 2021; Chen et al., 2021) to ensure the consistency of their experimental conditions. Figure 1 illustrates the basic structural features of these 69 inhibitor molecules, and we have selected eight representative molecules to be listed in Table 1.

**TABLE 1:** Structure of some representative inhibitors in the dataset.

No.	Structure	No.	Structure
5	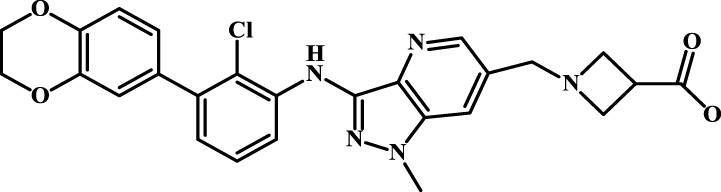	15	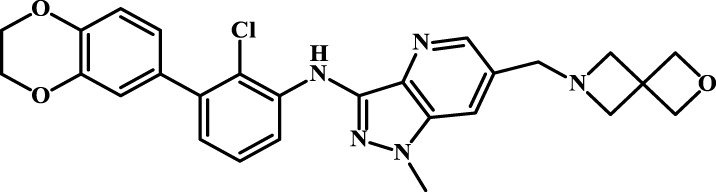
28	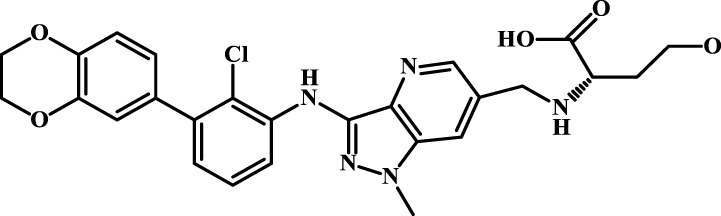	29	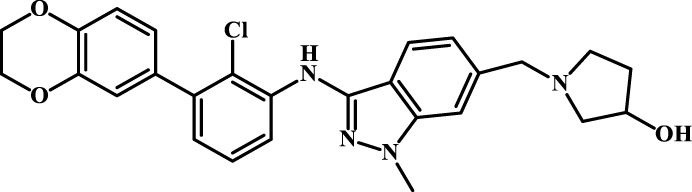
31	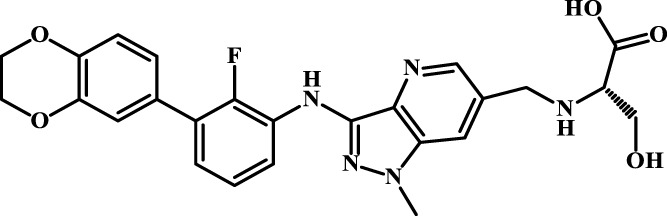	34	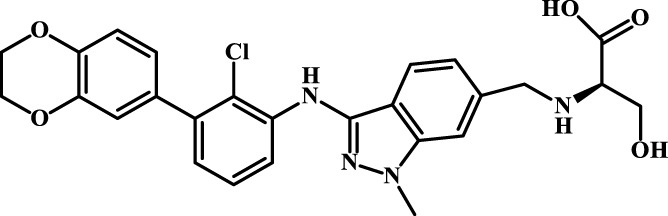
40	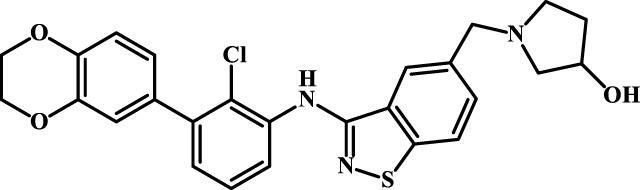	58	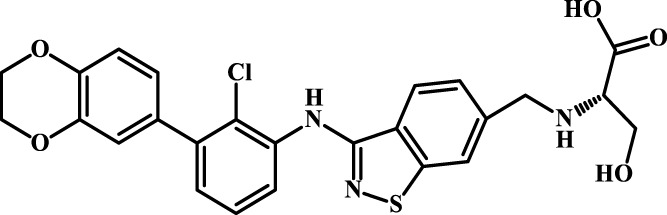

### 2.2 Molecular format and optimisation

The 2D structures of 69 molecules were drawn and saved in .cdx format using ChemDraw 2010 software, subsequently converted to 3D structures in .mol format and saved in .mol2 format. Based on the theory that lower energy molecules are more conformationally stable, the functional panel for energy minimisation in Sybyl software was used to hydrogenate all small molecules and optimize them using the Powell method. The maximum number of iterations was set to 1,000 and the gradient convergence criterion was set to the default of 0.05 kcal/mol/Å. Additional minimisation was performed using the Gasteiger-Huckel charge in the Tripos force field and all other settings were set to the default (Garcia et al., 2022; Chhatbar et al., 2019).

### 2.3 Molecular docking

To probe the binding mode of small-molecule ligands to protein macromolecules with the known 3D structures, GOLD molecular docking software was used and the scoring function GoldScore was chosen (Verdonk et al., 2003; Muszak et al., 2021). The crystal structure of the PD-L1 receptor (PDB ID: 6R3K) (Muszak et al., 2021) was obtained from the PDB database (http://www.rcsb.org/). The pocket used for docking is mainly composed of amino acids Ile54, Val55, Tyr56, Met115, Ile116, Ser117, Ala121, Asp122, Tyr123, Lys124. The number of dockings during the docking process was set to 10, and the rest were used as default values. All 69 molecules in the dataset were docked to the PD-L1 receptor. The best conformation of the molecules was determined based on docking scoring and the interaction between these inhibitors and the PD-L1 receptor was analysed. After docking, we determined the best conformation of the molecules based on the docking score and we also analysed and summarised the interactions between these inhibitors and the PD-L1 receptor for further study and analysis.

### 2.4 Synthesis and activity evaluation of small molecule inhibitors

The four novel PD-L1 inhibitors designed in this paper based on computational chemistry were all provided by Prof. Wang Yonghua’s group at Northwest A&S University (Ru et al., 2014) (95% purity). The biological activity of the compounds in blocking PDL1 binding was validated using an ELISA kit (ReagentGenie, UNFI0082). The basic principle is to coat a 96-well plate with human programmed death factor 1 and then add PD-L1 and the compound to be tested (i.e., a synthetic small molecule) to the microplate. The shade of colour is proportional to the amount of PD-L1. When the tested compounds interfere with the binding of PD-1 to PD-L1, the amount of PD-L1 bound to the monoclonal antibody decreases and the colour of the microtiter becomes lighter.

## 3 Results

### 3.1 Basic structural features of PD-L1 protein

**FIGURE 2:**
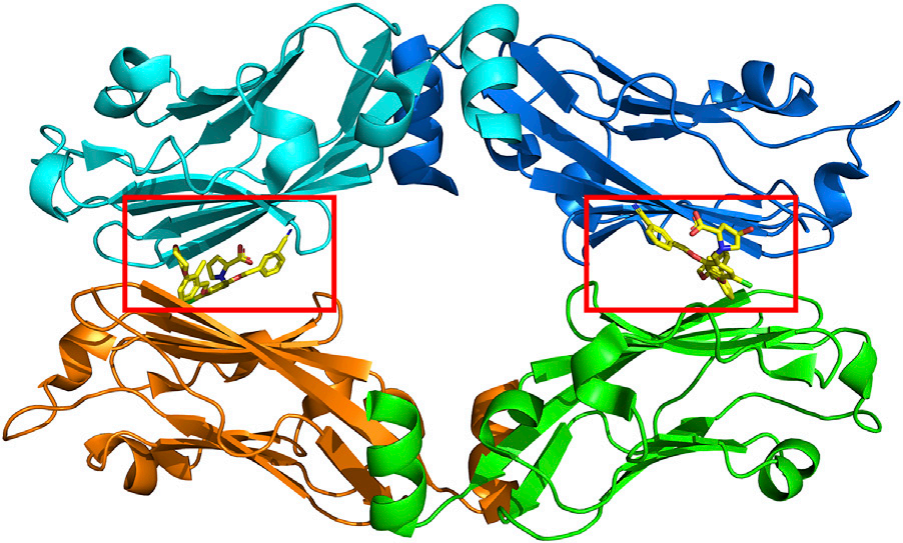
Three-dimensional structure of PD-L1 protein (PDB ID:6R3K).

PD-L1 is made up of two PD-L1 proteins, with two chains in each PD-L1 protein, with the same 128 amino acids present in each chain. These four chains are distinguished using four different colours in Figure 2. The red areas in the diagram indicate the presence of inhibitor small molecules in each PD-L1 protein. In addition, the two inhibitors are structurally and conformationally identical, and the space they occupy is the active site of the PD-L1 protein, also called the active cavity (Sobral et al., 2023; Aziz et al., 2023).

**FIGURE 3:**
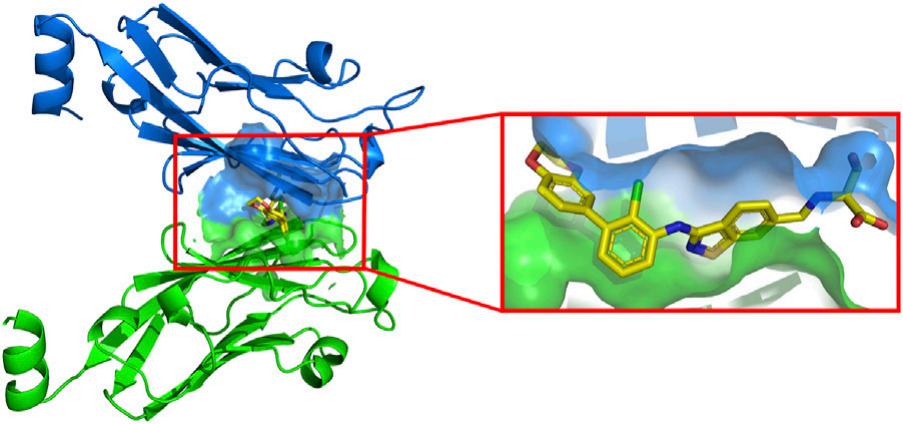
The active cavity of PD-L1 protein.

For ease of calculation, we have only studied the PD-L1 protein. There is an active cavity between two identical chains that allows small molecule inhibitors to bind to the protein, surrounded by a blue and green surface in Figure 3. The cavity is similar in shape to a “channel”, a narrow region with two open sides. The amino acid composition of this cavity is mainly Ile54, Val55, Tyr56, Met115, Ile116, Ser117, Ala121, Asp122, Tyr123, and Lys124. Small-molecule ligands can be stabilised in the cavity by various forces with the receptor. The inhibitor shown in the box is the most active molecule in the data set of this study, and we can see that the inhibitor shows a linear distribution of active conformations in the cavity.

### 3.2 Molecular docking scoring results

Molecular docking is a common method to study the interaction between small-molecule ligands and large molecule receptors. In the present study, we collected a total of 69 inhibitors of PD-L1 protein, all of which originated from the same laboratory and shared a similar backbone. In order to reveal the binding conformation and corresponding mechanism of action of these backbone inhibitors with PD-L1 protein, we performed molecular docking on all 69 molecules and recorded the docking scoring results. The number of dockings for each inhibitor molecule was set to 10 and the scoring function was chosen as GoldScore, with the docking results taken to two decimal places. Each docking score exceeded 70, with an average docking score of 77.61, indicating that each inhibitor has a high affinity for the PD-L1 protein receptor in Table 2.

**TABLE 2:** Summary of the GoldScore of 69 molecules in the dataset.

No.	GoldScore	No.	GoldScore	No.	GoldScore
1	81.74	2	76.44	3	75.18
4	75.21	5	72.66	6	72.73
7	80.32	8	76.42	9	77.40
10	78.01	11	74.04	12	76.95
13	73.69	14	75.37	15	75.02
16	73.89	17	75.6	18	75.13
19	77.75	20	75.71	21	76.81
22	76.42	23	79.29	24	74.63
25	77.92	26	76.12	27	78.96
28	77.11	29	74.33	30	76.46
31	72.67	32	70.50	33	80.28
34	74.98	35	74.04	36	72.63
37	75.80	38	79.81	39	79.75
40	76.03	41	79.26	42	81.22
43	76.66	44	80.80	45	78.33
46	74.06	47	75.85	48	73.27
49	76.39	50	76.78	51	81.15
52	77.52	53	83.62	54	80.42
55	77.24	56	78.81	57	78.09
58	84.63	59	83.14	60	81.68
61	87.02	62	76.79	63	79.86
64	78.04	65	81.75	66	82.89
67	82.45	68	82.64	69	81.12

### 3.3 Effect of substituents on the active conformation

**FIGURE 4:**
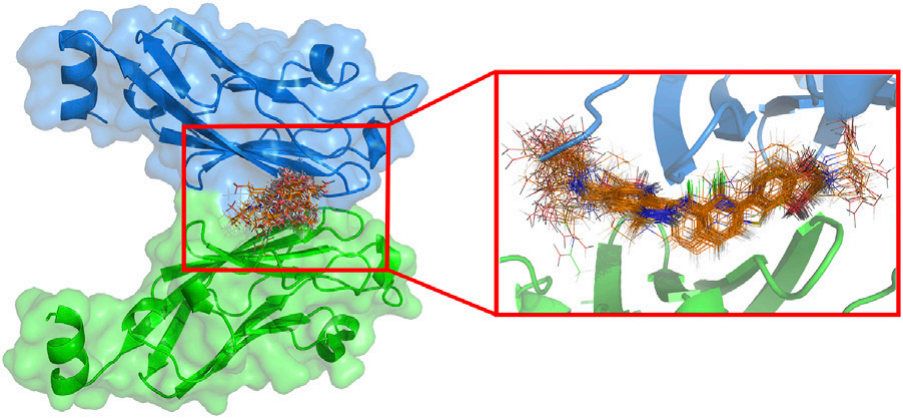
The superposition of active conformation of 69 molecules in the active cavity.

The difference in substituents during docking of these inhibitors with similar backbones may result in some variation in the active conformation of the inhibitor within the active cavity, and also have an impact on the docking effect. To investigate the overall differences in the active conformation of all molecules in the cavity in the dataset, the docking results were superimposed in Figure 4. 69 inhibitors were found to enter the same cavity. Overall, the conformations of the molecules are linear, occupying the entire cavity of the PD-L1 protein and are stable. The figure shows that the docking conformation of the backbone portion of these molecules stacked well, but differed considerably at the R1 and R3 substituents.

With a high degree of conformational similarity in the backbone, the main factors affecting the molecular activity and active conformation are the forces between the different substituents and the acceptor, including salt bridges, electrostatic forces, hydrophobic interactions, etc. The most common and most influential force is hydrogen bonding. We generally considered hydrogen bonds with bond lengths less than 3.5 Å to be strong, and bonds between 3.2 Å and 4.0 Å to be weak (Acurcio et al., 2018). Of these docking results, the molecules with the highest and second-highest activity rankings were selected for detailed study and analysis to investigate the mechanism of action of these inhibitors in binding to PD-L1 protein.

#### 3.3.1 Docking results for the most active molecule

**FIGURE 5:**
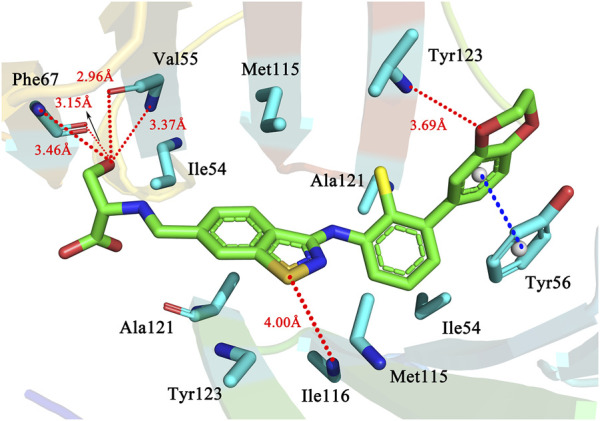
The docking results of the molecule 58 as PD-L1 inhibitor.

We first selected the most active molecule 58 for detailed description. The residues are all within 4.5 Å of the ligand and are mostly the amino acids that make up the active cavity in Figure 5. The ligand forms hydrogen bonding forces with a number of important surrounding amino acid residues (red dashed lines), which play an important role in stabilising the conformation and interaction of the inhibitor molecule within the active cavity. The sulphur atom of the Y-substituted part of the common backbone acts as a hydrogen bond acceptor and Ile116 as a hydrogen bond donor, forming a hydrogen bond (S … HN, 4.00 Å) that allows the backbone part of molecule 58 to be stabilised in the cavity. The R1 substituent is a 2,3-dihydro-1,4-benzodioxane group, which has a bicyclic structure. On the one hand, where the benzene ring forms a π-π stacking interaction with the benzene ring in the Tyr56 residue (blue dashed line) at a distance of 3.91 Å. On the other hand, an oxygen atom on the heterocyclic ring acts as a hydrogen bond acceptor and Tyr123 as a hydrogen bond donor, thus forming a hydrogen bond (O … HN, 3.69 Å). The stable conformation of the R1 substituent in the cavity results from the combination of the two forces mentioned above. At the same time, several hydrogen bonding forces are found at the R3 substituent. It is the synergistic effect of the various forces, especially hydrogen bonding, that positively influences the inhibition of PD-L1 protein activity by the molecule 58.

The carbon atoms of molecule 58 and residues are represented in green and blue, Nitrogen, oxygen, sulfur, and chlorine atoms are represented in mazarine, red, orange, and yellow, respectively. The red dashed lines indicate the H-bonds, and the blue dashed lines indicate the π-π stacking interactions.

#### 3.3.2 Docking results for the second reactive molecule 34

**FIGURE 6:**
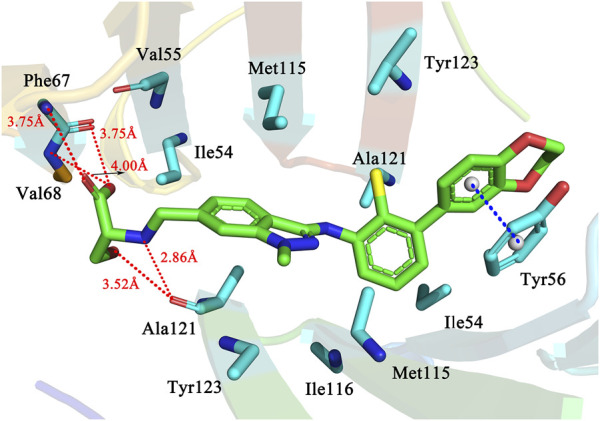
The docking results of molecule 34 as a PD-L1 inhibitor.

In addition to the most active molecule 58, the second active molecule 34 has been analysed in equal detail to investigate and examine the mechanism of their different activities. The nitrogen atom on the R3 substituent acts as the hydrogen bond donor and the oxygen atom on Ala121 as the hydrogen bond acceptor, resulting in a strong hydrogen bond (NH … O, 2.86 Å), while the hydroxyl oxygen on the R3 substituent also acts as the hydrogen bond donor and forms another hydrogen bond with Ala121 (OH … O, 3.52 Å). At the same time, the carbonyl oxygen of the R3 substituent forms a hydrogen bond with Phe67 as a hydrogen bond acceptor (O … HN, 3.75 Å) and the oxygen atom on the carboxyl group forms two hydrogen bonds with Phe67 (OH … O, 3.75 Å) and Val68 (OH … N, 4.00 Å) respectively as a hydrogen bond donor. Conformation in the cavity. Similar to #58, molecule #34 also forms a π-π stacking interaction with the benzene ring in the Tyr56 residue at a distance of 3.67 Å, indicated by the blue dashed line. We also found a series of hydrophobic interactions between the small molecule and the receptor in Figure 6. In summary, there are more forces between the R3 substituent of molecule 34 and the receptor, and these forces have a greater impact on the inhibitory activity of the molecule 34.

The carbon atoms of the molecule 34 and residues are represented in green and blue, Nitrogen, oxygen, sulfur, and chlorine atoms are represented in mazarine, red, orange, and yellow, respectively. The red dashed lines indicate the H-bonds, and the blue dashed lines indicate the π-π stacking interactions.

### 3.4 Effect of hydrogen bonding on the active conformation

Analysis of the docking results of the top two active inhibitors in the above dataset showed that hydrogen bonding plays an important role in their presence in the active cavity of PD-L1 protein, mainly by stabilising the backbone and substituents of the inhibitor, thus exerting a large effect on the active conformation of the ligand. The generation of hydrogen bonding forces depends in large part on the amino acid residues surrounding the ligand. Therefore, to identify the key amino acid residues that contribute to the hydrogen bonding force, we not only docked all 69 molecules in the dataset but also analysed the hydrogen bonds formed between each inhibitor and the receptor protein. Finally, the frequency of hydrogen bond-forming residues was counted to determine which residues play a decisive role in the active conformation of such backbone inhibitors. The docking results for the selection of more representative inhibitor molecules showed in Table 3.

**TABLE 3:** Representative docking results in the dataset.

No.	GoldScore	Crucial residues	H-bond	Distance (Å)	Angle (°)
14	75.37	Thr20	-OH … O	3.42	168.0
		Tyr123	-N … HN	3.50	129.1
		Met115	-NH … S	3.98	103.8
		Ile116	-Cl … NH	3.93	107.6
15	75.02	Thr20	-O … HO	3.17	123.4
		Met115	-NH … S	3.90	103.9
		Tyr123	-N … HN	3.46	121.3
		Tyr123	-O … HN	2.98	91.1
25	77.92	Thr20	-O … HO	3.74	127.8
		Gln66	-NH … N	3.81	138.3
		Tyr123	-N … HN	3.95	130.4
28	77.01	Gln66	-O … HN	3.05	131.1
		Gln66	-NH … O	3.71	147.3
		Met115	-NH … S	3.88	107.1
		Ile116	-Cl … HN	3.74	102.4
		Tyr123	-N … HN	3.49	120.0
		Tyr123	-NH … O	4.00	92.7
35	74.04	Thr20	-O … HO	3.44	138.9
		Gln66	-O … HN	3.69	95.3
		Gln66	-OH … N	3.81	119.1
		Met115	-NH … S	3.94	137.2
		Asp122	-NH … O	3.18	124.6
		Asp122	-NH … O	3.93	127.5
		Tyr123	-N … HN	3.81	126.6

By analysing and summarising the docking results of 69 molecules in the dataset, we summarised all the residues that form hydrogen bonds and counted their frequency of occurrence. These residues were Tyr123 (44 times), Gln66 (28 times), Thr20 (28 times), Met115 (24 times), Asp122 (18 times) and Ile116 (17 times) in descending order of frequency. The analysis of the high frequency of these residues will help us to apply the receptor-based structure approach to rationalize and optimize the structure of new drug molecules, so that the inhibitor can better bind to the receptor PD-L1 protein, thus theoretically enhancing the biological activity of the inhibitor.

### 3.5 Design of novel PD-1/PD-L1 inhibitors and activity evaluation

This type of backbone inhibitor molecule has many heteroatoms, both in the backbone and in the three substituent parts, which can act as both hydrogen bond donors and hydrogen bond acceptors when forming hydrogen bonds with some atoms or functional groups acting in both roles. This makes it easier for the inhibitor to form hydrogen bonds with the surrounding neighbouring amino acid residues. The formation of hydrogen bonds can, to some extent, determine or influence the docking effect of the ligand to the acceptor protein, which in turn may affect the biological activity of the inhibitor.

We designed four novel PD-1/PD-L1 inhibitors using the most active molecule 58 in the dataset, as the template for structural modification in Table 4. The activity was evaluated by ELISA and the results suggested that the N2 molecule was inhibited by up to 68.53%.

**TABLE 4:** Four novel designs of PD-L1 inhibitors and activity evaluation.

Compound	Structure	Inhibition rate at 5 μM
N1	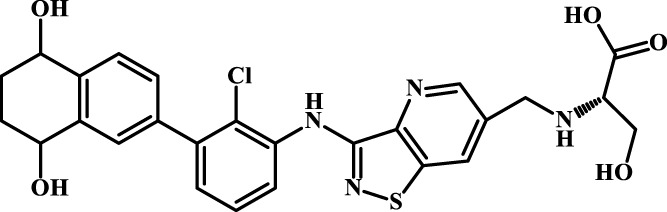	34.29%
N2	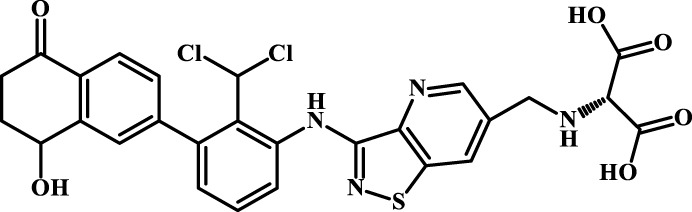	68.53%
N3	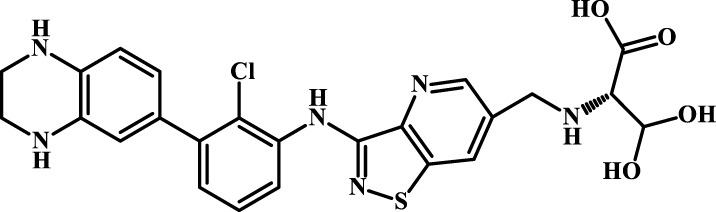	49.35%
N4	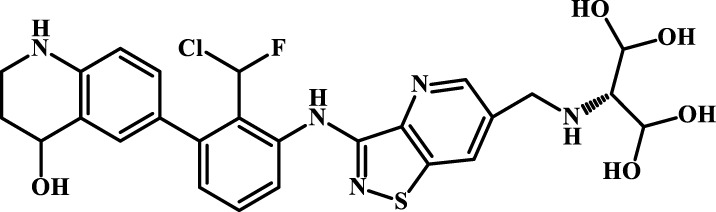	45.08%

## 4 Discussion

The PD-1/PD-L1 signalling pathway negatively regulates immune system T lymphocytes and plays an important role in inducing differentiation of effector T cells towards regulatory T cells, inhibiting T cell activation and inducing apoptosis of T cells (Lin et al., 2020). PD-1/PD-L1 The essence of immune checkpoint inhibitors is that they can inhibit or block the binding between PD-1 and PD-L1 to enable the body to recognize and kill cancer cells by reasserting immune function, thus achieving a therapeutic effect on cancer (Shen and Zhao, 2018; Yi et al., 2022). In this study, we used various methods to investigate the spatial structure of PD-1/PD-L1 and its key amino acids, explore the mechanism of action of PD-1/PD-L1 inhibitors, and to provide a reference for the development of novel PD-1/PD-L1 inhibitors.

From a detailed analysis of the docking results of the most active molecule 58 and molecule 34 in the above dataset, we found that although the two molecules are highly similar in structure and docking pattern, this may be an important reason why the two inhibitors possess different activities against the PD-L1 protein due to the subtle differences between the two in the backbone part and the R3 substituent. In the backbone part, the sulfur atom on the molecule 58 forms a weak hydrogen bond with the surrounding amino acid residue Ile116, resulting in a subtle difference from the backbone part of molecule 34, and preventing the nitrogen atom in the backbone part of molecule 34 from satisfying the distance condition for hydrogen bond formation with Ile116 due to the addition of the methyl group at the same position in molecule 34, increasing the spatial site resistance there. As far as the R3 substituent is concerned, the two are identical in composition, but the difference lies in the spatial orientation. The different spatial orientation makes it easier for molecule 34 to meet the distance requirement for hydrogen bonding with Ala121, and the presence of hydroxyl and carboxyl groups on the R3 substituent allows them to become hydrogen bond acceptors or hydrogen bond donors, respectively and thus form hydrogen bonds with the surrounding amino acid residues. As a result of docking, the hydroxyl group on the R3 substituent of molecule 58 and the carboxyl group on the R3 substituent of molecule 34 both enter a subcavity consisting of Ile54, Val55, Tyr56, Ile65, Gln66, Phe67 and Val68. Surrounding residues. Therefore, the main reason for the difference in activity between molecules 58 and 34 may attribute to the backbone portion and the R3 substituent, although structurally similar. The R1 substituent is not spatially oriented in the same way.

In general, when the nitrogen atom is in the X position is the backbone of the inhibitor molecule, it can easily form hydrogen bonds with Tyr123 as a hydrogen bond acceptor, which is also confirmed by our docking results. 76% (26/34) of the nitrogen atoms in this position in the data set formed at least one hydrogen bond with Tyr123, while at the same time the nitrogen atom could also form a hydrogen bond with Asp122, which could well stabilise the active conformation of the backbone part of the inhibitor in the cavity. On the other hand, Tyr123 forms the remaining hydrogen bond with the heteroatom on the R3 substituent of the inhibitor mainly as a hydrogen bond donor, which also plays a positive role in stabilising the conformation of the R3 substituent. There are many heteroatoms on the inhibitor R3 substituent, when these parts composed by the participation of heteroatoms enter the subcavity due to different spatial matching or spatial orientation, they can easily meet the distance and angle requirements for the formation of hydrogen bonds with the nitrogen or oxygen atoms on Gln66, Thr20 appears with a higher frequency similar to this. Through a large number of docking results, we found that the hydroxyl group on Thr20 is very close to the R3 substituent when the inhibitor is present in the active cavity, and the hydroxyl group can act as both a hydrogen bond acceptor and donor to form hydrogen bonding forces with the R3 substituent. In addition, the nitrogen atom on the R3 substituent also creates hydrogen bonds with the oxygen atom on Asp122. Similarly, the other substituents (R2 and R1) were analysed. Pharmacophore analysis and molecular dynamics simulations revealed that the design of PD-1/PD-L1 small molecule inhibitors needs to include the following structural features: (Heinzerling et al., 2021): a nitrogen atom at the X position can better form hydrogen bonds with Asp122 and Tyr123, thus stabilising the inhibitor backbone with the R3 substituent; (Seguin et al., 2022); additional hydrogen bond donors at the R1 substituent, which can be associated with Val55, Tyr56 Asp122, Tyr123 and other amino acids to stabilise the R1 substituent confirmation; (Hamilton and Rath, 2019); the addition of other heteroatomic functional groups to the R2 substituent in addition to the halogen atoms to form hydrogen bonds with Ile116; (Zhou et al., 2021); the addition of heteroatoms and branched chains to the R3 substituent to allow them to enter the subcavity to form hydrogen bonds and better anchor the R3 substituent.

In summary, we have performed a comprehensive computational study of 69 PD-1/PD-L1 inhibitors that possess biological activity. By using a molecular docking approach, the interaction mechanisms of these compounds with target proteins were explored and the active cavity composition of PD-L1 proteins, as well as the active conformation of the inhibitors, were determined. The effects of different substituents and their forces on the spatial conformation and activity of the small molecule inhibitors were examined. Meanwhile, the binding modes and characteristics of small molecule inhibitors and PD-L1 receptor target proteins were identified, their binding mechanisms were explored and explained, and their biological activities were initially explored, which provided a strong guide for the experimental studies of new PD-1/PD-L1 inhibitors.

## Data Availability

The datasets presented in this article are not readily available because All data used to support this study are available from the corresponding authors upon request. Requests to access the datasets should be directed to Yangyang, yangnj09@163.com.
